# Changes of T lymphocyte subpopulations and their roles in predicting the risk of Parkinson’s disease

**DOI:** 10.1007/s00415-022-11190-z

**Published:** 2022-05-24

**Authors:** Yijing He, Kangwen Peng, Ruoyu Li, Zhuoyu Zhang, Lizhen Pan, Tianyu Zhang, Ao Lin, Ronghua Hong, Zhiyu Nie, Qiang Guan, Lingjing Jin

**Affiliations:** 1 Neurotoxin Research Center of Key Laboratory of Spine and Spinal Cord Injury Repair and Regeneration of Ministry of Education, Neurological Department of Tongji Hospital, School of Medicine, Tongji University, 389 Xincun Road, 200065 Shanghai, People’s Republic of China; 2 Department of Neurology and Neurological Rehabilitation, Shanghai Yangzhi Rehabilitation Hospital (Shanghai Sunshine Rehabilitation Center), School of Medicine, Tongji University, Shanghai, 200092 People’s Republic of China; 3 Shanghai Clinical Research Center for Aging and Medicine, Shanghai, 200040 People’s Republic of China

**Keywords:** Parkinson’s disease, T lymphocyte subpopulation, Naïve CD8^+^ T cells, Late-differentiated CD4^+^ T cells, Nomogram

## Abstract

T lymphocytes are involved in the pathogenesis of Parkinson’s disease (PD), while the heterogeneity of T-cell subpopulations remains elusive. In this study, we analyzed up to 22 subpopulations of T lymphocytes in 115 PD patients and 60 matched healthy controls (HC) using flow cytometry. We found that PD patients exhibited decreased naïve CD8^+^ T cells (CD3^+^ CD8^+^ CD45RA^+^ CD45RO^−^) and increased late-differentiated CD4^+^ T cells (CD3^+^ CD4^+^ CD28^−^ CD27^−^), compared to HC, which were not affected by anti-parkinsonism medication administration. The proportion of naïve CD8^+^ T cells in PD patients was positively correlated with their severity of autonomic dysfunction and psychiatric complications, but negatively associated with the severity of rapid eye movement and sleep behavior disorder. The proportion of late-differentiated CD4^+^ T cells was negatively correlated with the onset age of the disease. We further developed individualized PD risk prediction models with high reliability and accuracy on the base of the T lymphocyte subpopulations. These data suggest that peripheral cellular immunity is disturbed in PD patients, and changes in CD8^+^ T cells and late-differentiated CD4^+^ T cells are representative and significant. Therefore, we recommend naïve CD8 + and late-differentiated CD4^+^ T cells as candidates for multicentric clinical study and pathomechanism study of PD.

**Supplementary Information:**

The online version contains supplementary material available at 10.1007/s00415-022-11190-z.

## Background

Parkinson’s disease (PD) is a neurodegenerative disease characterized by progressive degeneration of dopaminergic neurons and aggregation of alpha-synuclein (α-syn) in the substantia nigra (SN), which subsequently results in motor impairments [1, 2]. Mounting pieces of evidence implicates neuroinflammation contributing to the pathogenesis of PD. As the major component of the adaptive immune system, abundant T cells appear in the post-mortem brain of PD patients [1], whereas few are present in normal brains[2]. Recent studies have revealed that T cells in PD patients can recognize the specific epitopes derived from α-syn [3, 4]and mediate subsequent robust autoimmune reactions [5–7]. Therefore, the characteristics of T cells in the peripheral blood may reflect the pathophysiological changes at the early stage of PD. The prediction and diagnosis of that disease may rely upon identifying T-cell subsets in individuals.

T lymphocytes consist of two major groups: the CD4^+^ and CD8^+^ populations. The CD4^+^ T cells “help” the activity of other cells, while the CD8^+^ T cells mediate cytotoxicity. Both CD4^+^ and CD8^+^ T populations can be subdivided into different subsets according to their surface cluster of differentiation molecules or various functions. Previous pieces of evidence have indicated that infiltrating T cells exert diverse effects on the pathogenesis of PD. In PD patients, the infiltration of nigral CD8^+^ T cells initiates and propagates the progression of neuronal death and synucleinopathy [8]. Moreover, deficiency of CD4^+^ T cells in mice prevents the dopaminergic cell loss post 1-methyl-4-phenyl-1,2,3,6-tetrahydropyridine (MPTP) administration or α-syn overexpression [1, 9]. However, adoptive transfer of CD3-activated regulatory T cells (Tregs) or specific T cells from copolymer-1-immunized mice into MPTP-treated mice reduced microglia activation and neuron loss in the nigrostriatal system [10]. Although the diverse roles of T cells in the pathogenesis of PD are examined, the heterogeneity of T-cell subpopulations remains elusive.

Impairment in lymphatic vessels or blood–brain barrier was observed in PD [11, 12], which may suggest that intracephalic T-cell distribution is a reflection of that in peripheral blood. Several clinical investigations revealed that in peripheral blood, PD patients have fewer CD3^+^ and CD4^+^ T lymphocytes [13–15], but more Th17 cells [13, 16] compared to healthy controls (HC). Some studies reported a decrease in naïve T cells [17], Tregs [14, 18, 19], Th1 [20], and CD8^+^ T cells [18], whereas other studies show an increase in activated T cells [21] and CD8^+^ T cells [22] in PD patients. Due to relatively small sample sizes, different phenotyping methods, and patients’ recruitment, no consistent conclusion has been drawn so far.

In this study, we devote to uncovering the key T lymphocyte subpopulations associated with PD. To determine the relationship between the various symptoms of PD patients and the specific pattern of T lymphocyte subpopulations in PD condition, we profiled the clinical information of 115 PD patients and 60 HC individuals, and examined 22 T-cell subsets in peripheral blood. We found that PD patients exhibited less naïve CD8^+^ T (CD8^+^ Tn) cells and more late-differentiated (LD) CD4^+^ T cells. Based on our data, we generated nomogram models to predict the PD risk in individuals.

## Materials

### Participants

Participants were recruited from the Movement Disorder Outpatient of Tongji hospital from Nov 2017 to May 2020. Inclusion criteria: (i) patients with Parkinsonian symptoms aged 40–80 years who met the Movement Disorders Society (MDS) clinical diagnostic criteria for PD [23], diagnosed as clinically established PD or clinically probable PD; (ii) patients who presented modified Hoehn and Yahr (H&Y) rating scale from 1 to 3 stage; (iii) patients could cooperate in completing all the clinical evaluations; (iv) If the anti-parkinsonism medication was taken, the medication must be stable for 8 weeks before baseline. Exclusion criteria: (i) patients had a diagnosis of atypical parkinsonism or other central nervous system diseases; (ii) subjects whose CRP and/or WBC counts were above the upper limit of the normal range (upper limit of the normal range, CRP: 10 mg/L; WBCs: 10 × 10^9^/L); (iii) subjects with autoimmune disease or chronic infections, a history of immunosuppressive treatment and those on anti-inflammatory therapy such as NSAIDs; (iv) subjects were unable to cooperate with the clinical evaluation due to cognitive impairment or clinically significant mood disorder; (v) patients had been taking anti-parkinsonism drugs for less than 8 weeks or had adjusted their dose of anti-parkinsonism drugs within 8 weeks before enrollment. Age- and sex-matched HC were also included at the same time at the same location.

Following enrollment, subjects underwent a complete neurological examination and provided information on their disease states, including treatment history, age of PD onset, course of the disease, and drug administration. The current dose of anti-parkinsonism drugs taken was converted to the levodopa-equivalent daily dose (LEDD). Scales evaluations were conducted by filling in various questionnaires covering demographic information and motor/non-motor features of PD, and supervised by a qualified movement disorder specialist. Motor symptoms such as bradykinesia, rigidity, tremor, postural problems, balance problems, and motor complications were evaluated by the Movement Disorder Society Unified Parkinson’s Disease Rating Scale (MDS-UPDRS), Berg Balance Scale (BBS), Mini version of Balance Evaluation Systems Test (Mini-BEST), and Short Parkinson’s Evaluation Scale/Scales for Outcomes in Parkinson’s disease (SPES/SCOPA). Non-motor symptoms consist of anosmia, constipation, sleep disorders, emotional disorder, cognitive deficiency, autonomic dysfunction, and psychiatric complications. These features were evaluated by the Non-motor symptom scale (NMSS), Hyposmia rating scale (HRS), Constipation Severity Instrument (CSI), Pittsburgh sleep quality index (PSQI), REM sleep behavior disorder screening questionnaire (RBDSQ), Epworth Sleeping Scale (ESS), Restless Leg Syndrome (RLS), Hospital Anxiety and Depression Scale (HAD)-Anxiety part (A)/ Depression part (D), 17-item Hamilton Depression Scale (HAMD-17), Mini-Mental State Examination (MMSE), Scales for Outcomes in Parkinson's disease for Autonomic symptoms (SCOPA-AUT), and Scales for Outcomes in Parkinson’s disease for Psychiatric Complications (SCOPA-PC). The life quality of PD patients was measured by 39-item Parkinson’s Disease Questionnaire (PDQ-39).

This study was approved by the Ethics Committee of Tongji Hospital of Tongji University (Approved ID: KYSB-2017-097). All the participants signed the informed consent before enrollment. The study was performed according to the Declaration of Helsinki and the relevant ethical guidelines for research on humans.

### Blood cell analysis and flow cytometric analysis

Peripheral venous blood samples were collected after a fasting night, between 6:00 a.m. and 9:00 a.m., in EDTA-coated tubes. To ensure processing efficiency, no more than two individual blood samples were collected per day. Leukocyte and total lymphocytes analyses were performed immediately after blood sample collection, and were completed by Sysmex-XN A1 automatic hematology analyzer (Sysmex, Kobe, Japan).

For lymphocyte subsets analysis, 2 ml aliquots of whole blood were prepared from each participant and processed immediately to ensure homogeneous treatment. Each sample was divided and every 50 ul aliquots were incubated with a cocktail of different four-color panels fluorescein-conjugated antibody (FITC-PE-PerCP-APC) for targeted immunocytes: B cells (CD3^− ^CD19^+^), natural killer (NK) cells (CD3^−^ CD16^+ ^CD56^+^), T cells (CD3^+^), and 22 types of T-cell subsets, including NKT cells (CD3^+^ CD16^+ ^CD56^+^), CD4^+^ T cells (CD3^+^ CD4^+^), CD4^+^ naïve T (Tn) cells (CD3^+ ^CD4^+^ CD45RA^+^ CD45RO^−^), CD4^+^ memory T (Tm) cells (CD3^+^ CD4^+^ CD45RA^−^ CD45RO^+^), CD4^+^ effector memory T cells (Tem) cells (CD3^+^ CD4^+^ CD45RO^+^ CD62L^−^), CD4^+^ central memory T (Tcm) cells (CD3^+^ CD4^+^ CD45RO^+^ CD62L^+^), CD8^+^ T cells (CD3^+ ^CD8^+^), CD8^+^ Tn cells (CD3^+ ^CD8^+^ CD45RA^+ ^CD45RO^−^), CD8^+^ Tm cells (CD3^+ ^CD8^+ ^CD45RA^−^ CD45RO^+^), CD8^+^Tem cells (CD3^+^ CD8^+ ^CD45RO^+ ^CD62L^−^), CD8^+ ^Tcm cells (CD3^+^ CD8^+ ^CD45RO^+ ^CD62L^+^), activated CD4^+^ T cells (CD38^+ ^HLA-DR^+ ^CD3^+ ^CD4^+^), activated CD8^+^ T cells (CD38^+ ^HLA-DR^+ ^CD3^+^ CD8^+^), CD4^+^ regulatory T (Treg) cells (CD4^+^ CD25^+^ CD127^−^), CD8^+^ Treg cells (CD8^+^ CD28^−^), early differentiated (ED) CD4^+^ T cells (CD3^+^ CD4^+ ^CD28^+^ CD27^+^), medium-differentiated (MD) CD4^+^ T cells (CD3^+^ CD4^+^ CD28^+^ CD27^−^), LD CD4^+^ T cells (CD3^+ ^CD4^+ ^CD28^−^ CD27^−^), ED CD8^+^ T cells (CD3^+^ CD8^+ ^CD28^+ ^CD27^+^), MD CD8^+^ T cells (CD3^+^ CD8^+^ CD28^+^ CD27^−^), LD CD8^+^ T cells (CD3^+ ^CD8^+ ^CD28^−^ CD27^−^), and senescent T cells (CD57^+^ CD3^+^). The markers used for each individual subset of T cells are also listed in Table 2. After 30 min in the dark at room temperature, erythrocytes were removed by lysis solution (BD, 349,202). Samples were then centrifuged, supernatants were removed, and cells were washed in phosphate-buffered saline (PBS) and resuspended in PBS. Antibodies used in the study are listed in Online Resource 1. Acquisition and data analysis were then performed on a BD Accuri C6 flow cytometer (Becton Dickinson, Milan, Italy) with BD C6 software (version 1.0.264.21). To ensure high-quality and consistent results, the flow cytometry instrument was calibrated daily, and antibody fluorescence intensity was monitored weekly.

Data were analyzed with the BD C6 software (version 1.0.264.21). Unstained samples were used to set voltages for fluorescence channels and single-color controls were set to correct for fluorescence spillover. The gating strategy for lymphocytes using side scatter (SSC) and forward scatter (FSC) according to Salzman et al. [24]. 5000 cells were obtained in the lymphocyte gate to make sure that the same number of cells was used for each sample. All percentage and statistical analyses were performed inside the lymphocytes gate, thus excluding cell debris. The isotype controls were set to inform the gating. The gating strategy is shown in Online Resource 2. The results are finally expressed as the percentage of positive cells (%) in the father group, and the father group is, respectively, remarked on the vertical coordinates of boxplots in Fig. 2.

### Statistical analysis

Data are expressed as mean (standard deviation range), median (interquartile range), or percentage depending on the type of data and their distribution. Continuous variables were subjected to Student’s *t* tests if they fit a normal distribution with homogeneous variances; otherwise, Mann–Whitney tests were used. Differences in categorical variables between groups were tested by the Chi-square test. Multivariate logistic regression analyses were applied to adjust for potential confounders and determine the factors affecting PD diagnosis. A nomogram based on the results of previous multivariable analyses was constructed to predict PD risk. The calibration curve was used to analyze the agreement between the nomogram and actual observation. Confidence intervals were obtained by creating 1000 bootstrap samples from the corresponding cohort and replicating the estimation process. Discrimination was assessed using the concordance index (C-index). Linear regression was used to identify major clinical features of PD patients in response to their lymphocyte changes. Statistical analyses were conducted using the SPSS version 19.0 software (IBM Corporation, Armonk, NY, USA) and R version 3.5.2 (http://www.r-project.org). *P* < 0.05 was regarded as statistically significant.

## Results

### Baseline characteristics

**Fig. 1:**
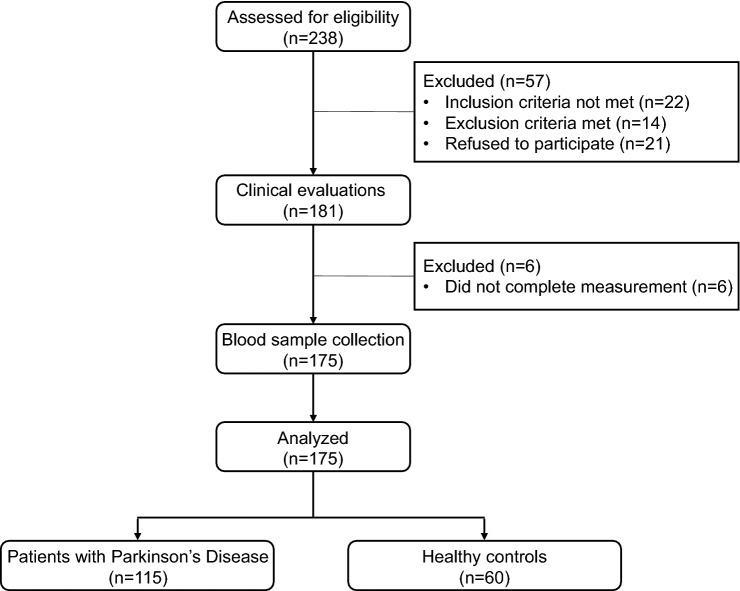
The flow diagram shows the process of the study

We recruited 238 participants in Movement Disorder Outpatient of Tongji hospital, and 63 of them were excluded for inclusion criteria not met (*n* = 22), exclusion criteria met (*n* = 14), refused to enroll (*n* = 21), and incomplete clinical evaluations (*n* = 6). Finally, a total of 115 PD patients entered the analysis. At the same time, 60 age- and sex-matched HCs were included in the analysis (Fig. 1). Generally, there was no significant difference in age, sex, and BMI between the PD patients and HC individuals. In terms of lifelong habits, more people in the HC group had habits of smoking, alcohol consumption, and tea consumption than that in the PD group, while the rate of coffee consumption and exercise showed no differences between the two groups. Interestingly, the comparison of past medical history between the two groups showed comorbidity that more people in the PD group suffered diabetes after adjusting for multiple covariates such as age, sex, smoking, and alcohol consumption (Table 1).

**Table 1:** Basic demographics, past medical history, and habits of enrollers

Basic demographics	HC	PD	*P* value
	*n* = 60	*n* = 115	
Age, (years, mean ± SD)	65.71 ± 5.35	63.40 ± 9.13	0.072
Gender, (females, %)	43.3	47	0.648
BMI, (kg/m^2^, mean ± SD)	23.86 ± 3.09	22.89 ± 3.37	0.063
Past medical history			
Hypertension, (%)	35	33.9	0.674^a^
Diabetes mellitus, (%)	8.3	13	0.043^a*^
Hyperlipidemia, (%)	28.3	16.5	0.251^a^
Traumatic brain injury, (%)	1.7	6.1	0.184
Pesticide exposure, (%)	1.7	7.8	0.096
Habits			
Smoking, (%)	13.3	3.5	0.014^*^
Alcohol consumption, (%)	41.7	25.2	0.025^*^
Tea consumption, (%)	55	34.8	0.010^*^
Caffeine consumption, (%)	31.7	24.3	0.300
Exercise, (%)	75	73	0.857

*P* values were calculated by Student's *t*-test (continuous variables) or the chi-square test (categorical variables)

*HC* healthy control, *PD* Parkinson’s disease, *BMI* Body Mass Index, *SD* standard deviation

^a^Indicates age, sex, BMI, smoking (%), and alcohol consumption (%) were adjusted when compared between/among groups. *indicates *P* < 0.05

### LD CD4^+^ T cells and CD8^+^ Tn cells are independent predictors of PD condition

**Fig. 2:**
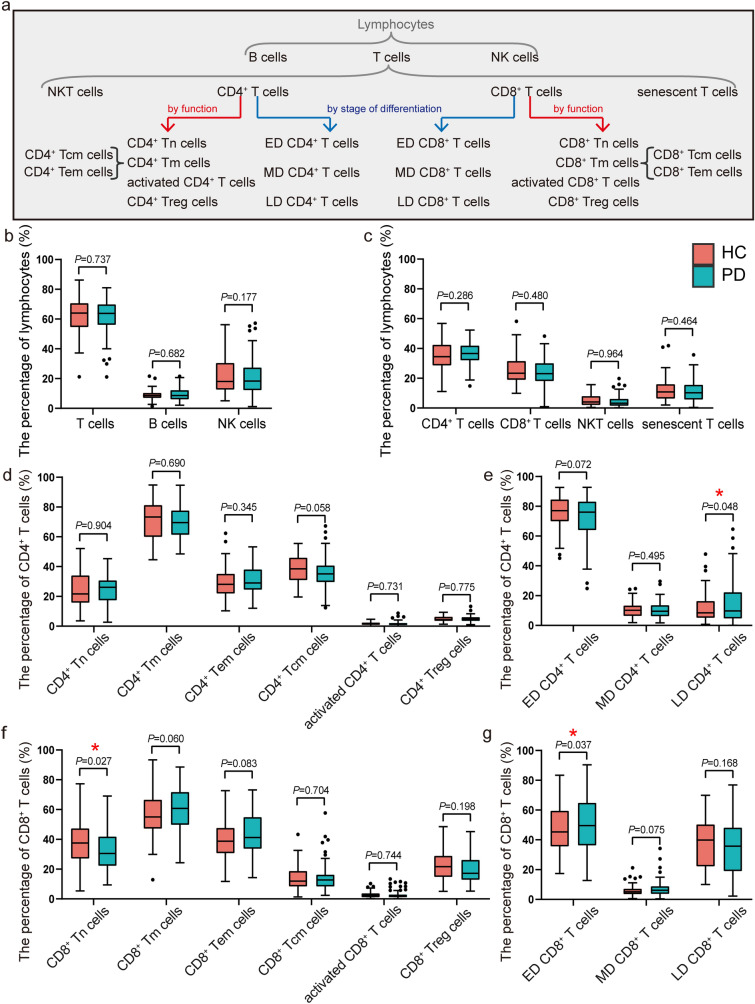
Level of lymphocyte subpopulations in peripheral blood between groups of patients with Parkinson’s disease and healthy controls. **a** Lymphocyte subpopulations classification strategy used in this study. **b** Distribution of the major lymphocyte subsets (T cells, B cells, NK cells). **c** Distribution of CD4^+^ T lymphocytes, CD8^+^ T lymphocytes, NKT cells, and senescent T cells. **d** Distribution of CD4^+^ T lymphocytes’ functional subpopulations (CD4^+^ Tn cells, CD4^+^ Tm cells, CD4^+ ^Tem cells, CD4^+^ Tcm cells, activated CD4^+^ T cells, and CD4^+^ Treg cells). **e** Distribution of CD4^+^ T cell differentiated subpopulations (ED CD4^+^ T cells, MD CD4^+^ T cells, LD CD4^+^ T cells). **f** Distribution of CD8^+^ T lymphocytes’ functional subpopulations (CD8^+^ Tn cells, CD8^+^ Tm cells, CD8^+^ Tem cells, CD8^+^ Tcm cells, activated CD8^+^ T cells and CD8^+^ Treg cells). **g** Distribution of CD8^+^ T cell differentiated subpopulations (ED CD8^+^ T cells, MD CD8^+^ T cells, LD CD8^+^ T cells). Data were presented as a box plot, with the center, box, whiskers, and points corresponding to the median, interquartile range, extremum range, and outliers, respectively. *P* values were calculated by Student’s t test (variables of CD8^+^ Tm cells and ED CD8^+^ T cells) or Mann–Whitney test (variables except for CD8^+^ Tm cells and ED CD8^+^ T cells). PD: *n* = 115, HC: *n* = 60. * indicates *P* < 0.05. *PD* Parkinson’s disease, *HC* Healthy control, *NK * Natural killer, *Tn* Naive T cells, *Tm* Memory T cells, *Tcm* Central Memory T cells, *Tem* Effector Memory T cells, *Treg* Regulatory T cells, *ED* Early differentiated, *MD* Medium-differentiated, *LD* Late-differentiated

We investigated the characteristics of T lymphocytes in peripheral blood of 115 PD patients and 60 HC persons. In general, the total lymphocytes descended in the PD group, including both proportion and absolute number levels compared with HC in our cohort (*P* < 0.05), while the number of leukocytes was similar between the two groups (Online Resource 3). To elucidate the precise changes of lymphocyte subpopulations in PD conditions, we next analyzed 25 types of lymphocytes, including 22 subtypes of T cells. The classifications are based on cell surface markers, focusing on functional and differentiated subpopulations. The distributions of each lymphocyte subpopulation in the PD and the HC groups are shown in Fig. 2. And the alteration of CD8^+^ Tn cells, LD CD4^+^ T cells, and ED CD8^+^ T cells in PD patients reached statistical differences (*P* < 0.05) in univariate analysis of cell proportions. Compared with HC subjects, LD CD4^+^ T cells and ED CD8^+^ T cells were upregulated but CD8^+^ Tn cells were downregulated in PD patients (Fig. 2), suggesting a redistribution of T lymphocyte subsets in PD condition.

Next, we conducted a logistic regression analysis based on univariate analysis, which shows that the proportion of CD8^+^ Tn cells (*P* < 0.05), LD CD4^+^ T cells (*P* < 0.05), and ED CD8^+^ T cells are associated with PD condition, after adjustment for confounding factors, such as diabetes mellitus status, smoking consumption, alcohol consumption, and tea consumption (Table 2). To further confirm the roles of these lymphocyte subsets in independent predictive for PD, we then built logistic regression models based on their absolute cell counts. The models established a firm position of CD8^+^ Tn cells (*P* < 0.05) and LD CD4^+^ T cells (*P* < 0.05) as independent correlation factors for PD, while the ED CD8^+^ T cells were eliminated in this model due to the unstable performance (Table 2).

**Table 2:** Logistic regression analysis of immunocyte predictors for the diagnosis of Parkinson’s disease in the cohort

Immunocyte (%)	Marker	Cell proportion		Absolute cell count	
		AOR [95% CI]	*P* value	AOR [95% CI]	*P* value
Leukocytes (× 10^12^/L)	–	–	–	1.110 [0.862, 1.428]	0.419
Lymphocytes (× 10^9^/L)	–	0.948 [0.905, 0.992]	0.021*	0.562 [0.293,1.080]	0.084
T cells (× 10^9^/L)	CD3^+^	0.998 [0.970, 1.027]	0.918	0.479 [0.199, 1.155]	0.101
B cells (× 10^6^/L)	CD3^− ^CD19^+^	1.010 [0.929, 1.098]	0.818	0.999 [0.995, 1.002]	0.426
NK cells (× 10^6^/L)	CD3^−^ CD(16^+ ^56)^+^	0.996 [0.967, 1.026]	0.790	0.999 [0.998, 1.001]	0.313
NKT cells (× 10^6^/L)	CD3^+^ CD(16^+^ 56)^+^	0.946 [0.868, 1.032]	0.213	0.996 [0.992, 1.001]	0.094
CD4^+^ T cells (× 10^6^/L)	CD3^+ ^CD4^+^	1.021 [0.982, 1.061]	0.300	0.999 [0.998, 1.001]	0.452
Functional Subgroups					
CD4^+^ Tn cells (× 10^6^/L)	CD3^+^ CD4^+^ CD45RA^+^ CD45RO^−^	0.999 [0.967, 1.033]	0.974	0.998 [0.995, 1.002]	0.318
CD4^+^ Tm cells (× 10^6^/L)	CD3^+^ CD4^+^ CD45RA^−^ CD45RO^+^	0.993 [0.965, 1.023]	0.656	0.999 [0.998, 1.001]	0.603
CD4^+^ Tem cells (× 10^6^/L)	CD3^+^ CD4^+ ^CD45RO^+ ^CD62L^−^	1.018 [0.982, 1.055]	0.341	1.001 [0.998, 1.005]	0.502
CD4^+^ Tcm cells (× 10^6^/L)	CD3^+^ CD4^+ ^CD45RO^+^ CD62L^+^	0.963 [0.928, 0.999]	0.043*	0.997 [0.994, 1.000]	0.079
Activated CD4^+^ T cells (× 10^6^/L)	CD38^+ ^HLA-DR^+^ CD3^+^ CD4^+^	0.957 [0.741, 1.238]	0.740	0.994 [0.967, 1.032]	0.745
CD4^+^ Treg cells (× 10^6^/L)	CD4^+^ CD25^+ ^CD127^−^	1.015 [0.847, 1.217]	0.873	0.997 [0.973, 1.022]	0.804
Differentiated subgroups					
ED CD4^+^ T cells (× 10^6^/L)	CD3^+ ^CD4^+^ CD28^+ ^CD27^+^	0.974 [0.950, 1.000]	0.048*	0.998 [0.997, 1.000]	0.052
MD CD4^+^ T cells (× 10^6^/L)	CD3^+ ^CD4^+^ CD28^+ ^CD27^−^	0.977 [0.917, 1.041]	0.474	0.997 [0.988, 1.006]	0.507
LD CD4^+^ T cells (× 10^6^/L)	CD3^+^ CD4^+^ CD28^−^ CD27^−^	1.032 [1.003, 1.063]	0.031*	1.004 [1.000, 1.008]	0.028*
CD8^+^ T cells (× 106/L)	CD3^+ ^CD8^+^	0.986 [0.950, 1.023]	0.446	0.999 [0.997, 1.000]	0.097
Functional subgroups					
CD8^+^ Tn cells (× 10^6^/L)	CD3^+^ CD8^+ ^CD45RA^+ ^CD45RO^−^	0.971 [0.946, 0.996]	0.021*	0.996 [0.993, 0.999]	0.024*
CD8^+^ Tm cells (× 10^6^/L)	CD3^+^ CD8^+^ CD45RA^−^ CD45RO^+^	1.023 [1.000, 1.047]	0.048*	0.999 [0.997, 1.001]	0.510
CD8^+^ Tem cells (× 10^6^/L)	CD3^+^ CD8^+ ^CD45RO^+ ^CD62L^−^	1.025 [0.998, 1.053]	0.070	0.999 [0.996, 1.002]	0.562
CD8^+^ Tcm cells (× 10^6^/L)	CD3^+ ^CD8^+ ^CD45RO^+^ CD62L^+^	0.993 [0.954, 1.034]	0.744	0.996 [0.989, 1.003]	0.238
Activated CD8^+^ T cells (× 10^6^/L)	CD38^+ ^HLA-DR^+^ CD3^+^ CD8^+^	0.980 [0.858, 1.119]	0.766	0.981 [0.954, 1.009]	0.181
CD8^+^ Treg cells (× 10^6^/L)	CD8^+ ^CD28^−^	0.977 [0.944, 1.011]	0.180	0.998 [0.996, 0.999]	0.009**
Differentiated subgroups					
ED CD8^+^ T cell (× 10^6^/L)	CD3^+ ^CD8^+ ^CD28^+^ CD27^+^	1.023 [1.003, 1.043]	0.027*	1.001 [0.998, 1.005]	0.417
MD CD8^+^ T cell (× 10^6^/L)	CD3^+ ^CD8^+ ^CD28^+^ CD27^−^	1.087 [0.994, 1.189]	0.066	1.007 [0.991, 1.023]	0.379
LD CD8^+^ T cell (× 10^6^/L)	CD3^+^ CD8^+^ CD28^− ^CD27^−^	0.986 [0.968, 1.005]	0.141	0.997 [0.995, 1.000]	0.022*
Senescent T cell (× 10^6^/L)	CD57^+^ CD3^+^	0.984 [0.941, 1.028]	0.463	0.999 [0.996, 1.001]	0.182

*HC* healthy control, *PD* Parkinson’s disease, *H&Y* Hoehn and Yahr rating scale, *NK * natural killer, *Tn* naive T cells, *Tm* memory T cells, *Tcm* central memory T cells, *Tem* effector Memory T cells, *Treg* regulatory T cells, *ED* early-differentiated, *MD* medium-differentiated, *LD* late-differentiated, *AOR* adjusted odds ratio, *CI* confidence interval

*P* values were calculated by logistic regression after adjusting factors included diabetes mellitus (%), smoking (%), alcohol consumption (%), and tea consumption (%). *Indicates *P* < 0.05, **indicates *P* < 0.01

To clarify whether this cellular discrepancy is due to medication administration in PD patients, we further analyzed the peripheral lymphocyte characteristics of drug-naïve patients in the cohort versus those who had been treated with stable medication for 8 weeks. The results showed that the proportion of CD8^+^ Tn cells and the proportion of LD CD4^+^ T cells did not differ between those two groups. Moreover, the changes in cell numbers and their proportions shared a similar trend (Online Resource 4). Our data suggest that CD8^+^ Tn cells and LD CD4^+^ T cells are potentially critical factors for PD prediction.

### The association between T lymphocyte subset indicators and clinical characteristics of PD

**Fig. 3:**
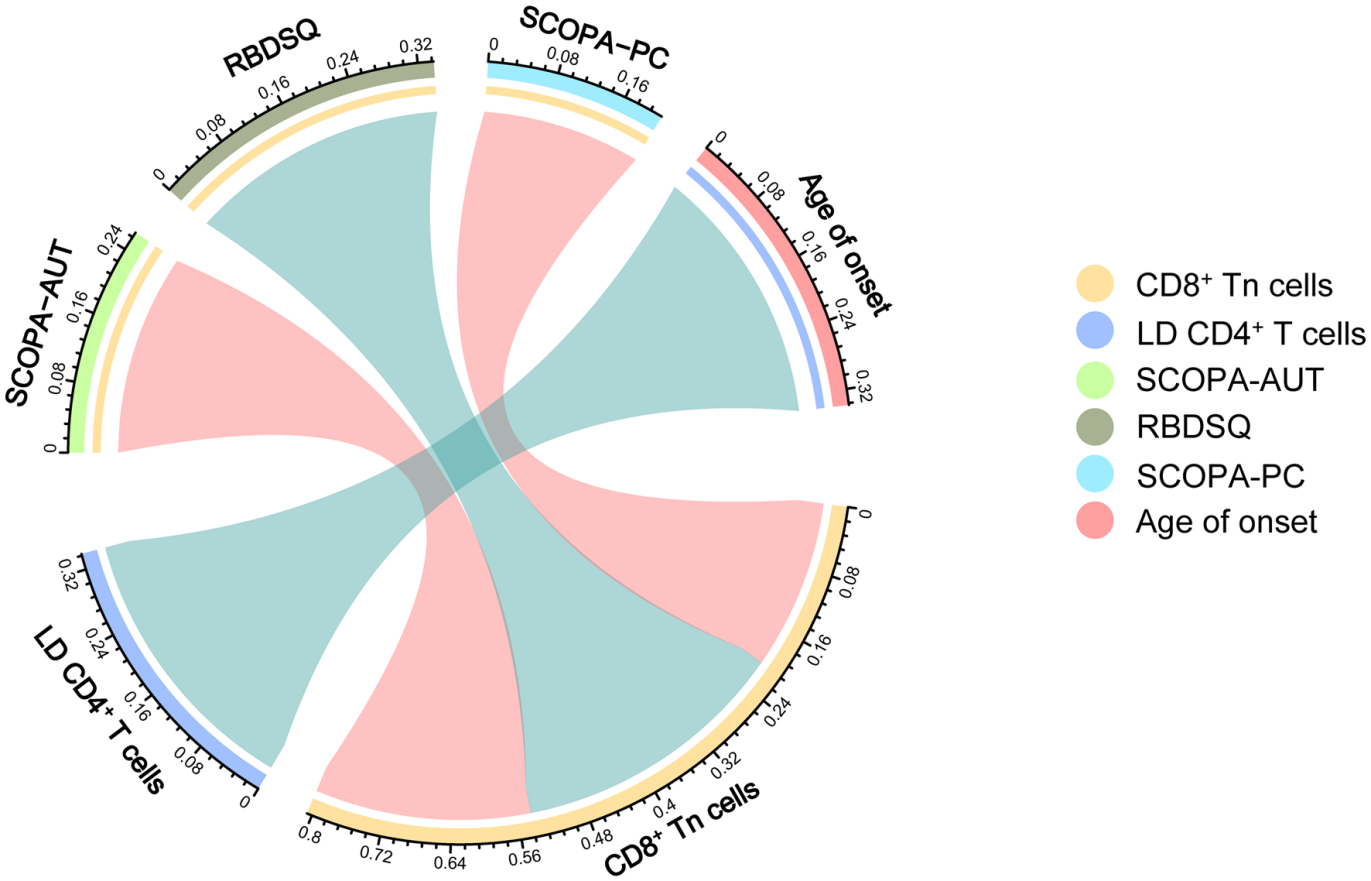
The chord diagram shows the normalized coefficients among lymphocyte subpopulations and indicators associated with Parkinson’s disease. The red line represents a positive correlation between the percentage of lymphocyte subsets in the blood and the severity of non-motor symptoms, while the green line represents a negative correlation between the percentage of lymphocyte subsets and the severity of non-motor symptoms or age at onset. The width of the connecting line represents the normalized correlation coefficient. *P* < 0.05 was regarded as statistically significant. *PD* Parkinson’s disease, *CD8*^+^ *Tn cells* naïve CD8^+^ T cells, *LD CD4*^+^ *T cells* late-differentiated CD4^+^ T cells, *SCOPA-AUT* Scales for outcomes in Parkinson’s disease for autonomic symptoms, *RBDSQ* REM sleep behavior disorder screening questionnaire, *SCOPA-PC* Scales for outcomes in Parkinson’s disease for psychiatric complications

To further determine the relevant clinical features of CD8^+^ Tn cells and LD CD4^+^ T cells, we used multiple regression models to explore the association between their proportions and characteristics of PD patients (age of disease onset, course of the disease, LEDD, H&Y, scores of motor symptoms scales included MDS-UPDRS, BBS, Mini-BEST, SPES/SCOPA, scores of non-motor symptoms included NMSS, HRS, CSI, PSQI, RBDSQ, ESS, RLS, HAD, HAMD-17, MMSE, SCOPA-AUT, SCOPA-PC, and scores of life quality from PDQ-39). All the features of our PD patients are summarized in Online Resource 5. We found that the proportion of CD8^+^ Tn cells in peripheral blood were positively correlated with severity of autonomic dysfunction (SCOPA-AUT) (correlation coefficient 0.398, 95% CI 0.119–0.676, *P* = 0.006) and psychiatric complications (SCOPA-PC) (correlation coefficient 2.883, 95% CI 0.179–5.587, *P* = 0.037), and negatively associated with severity of RBD (RBDSQ) (correlation coefficient − 1.193, 95% CI − 1.886 to − 0.501, *P* = 0.001) (adjusted *R*-squared 0.118, *F* = 6.061, *P* = 0.001). And LD CD4^+^ T cells are negatively correlated with age of disease onset (correlation coefficient -0.428, 95% CI − 0.650 to − 0.206, *P* < 0.001) (adjusted *R*-squared 0.106, *F* = 14.563, *P* < 0.001) (Fig. 3). These results indicated that clinical phenotyping connects with cellular change, which further exhibited the possibility of using CD8^+^ Tn cells and LD CD4^+^ T cells as predictors of PD condition.

### Development of nomograms to assess the relationship between T lymphocyte subsets and PD risk

**Fig. 4:**
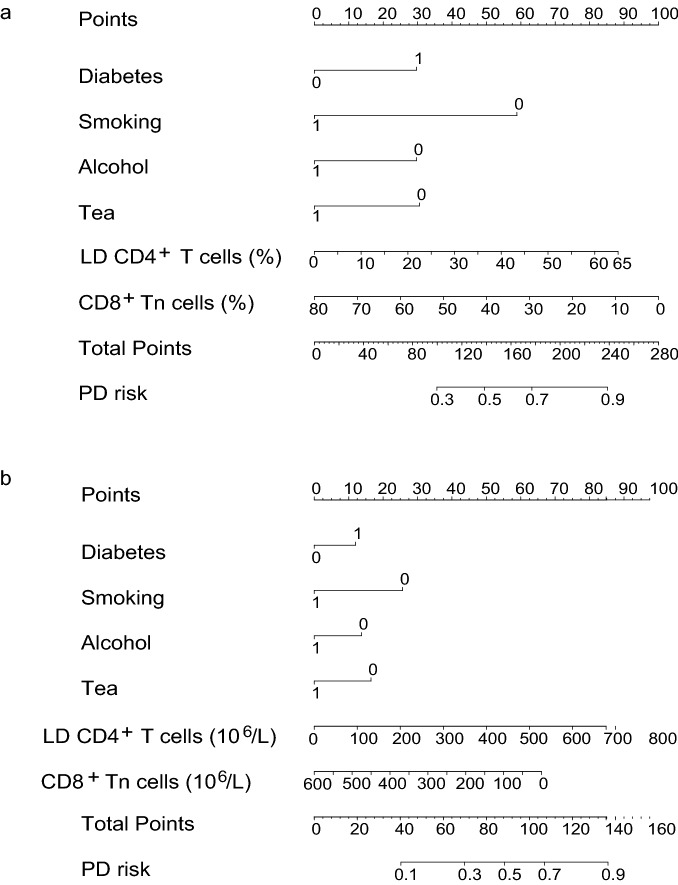
Nomograms for calculating risk score of Parkinson’s disease in aged 40–80 year subjects. The predictive nomograms for PD risk are developed with diabetes, smoking, alcohol consumption, tea consumption, LD CD4^+^ T cells, and CD8^+^ Tn cells. a. The variables of LD CD4^+^ T cells and CD8^+^ Tn cells show in **a** the percentage of LD CD4^+^ T cells in CD4^+^ T cells and the percentage of CD8^+^ Tn cells in CD8^+^ T cells; **b** their absolute cells number in peripheral blood. Weight points were assigned for six variables by the vertical line between the top points scale and each variable. The sum of all these scores, plotted on the ‘Total point’ line, corresponds to predictions of PD occurrence probability in aged 40–80 year subjects. *PD* Parkinson’s disease, *CD8*^+^ *Tn cells* naïve CD8^+^ T cells, *LD CD4*^+^ *T cells* late-differentiated CD4^+^ T cells

To quantify the contribution of T lymphocyte subsets in increasing the risk of PD, we developed the nomogram for PD risk prediction by assigning a weighted point to each of the independent risk factors, including the level of CD8^+^ Tn cells, level of LD CD4^+^ T cells, and status of diabetes mellitus, smoking, alcohol consumption, and tea consumption. For clinical application, two separate models were constructed for cell proportions and absolute cell counts, respectively. The highest total score of the nomogram contained the cell proportions of 280 points, and the scale of PD risk probability ranged from 0.3 to 0.9. The highest total score of the nomogram contained the cell counts of 160 points, and the scale of PD risk probability ranged from 0.1 to 0.9. A higher total score calculated from the sum of the assigned points for each predictive factor in the nomogram corresponded to a higher risk for the development of PD (Fig. 4). The exact nomogram scores were recorded in Online Resource 6. These models can be developed as visual tools for identifying PD patients who are clinically difficult to diagnose by symptoms.

**Fig. 5:**
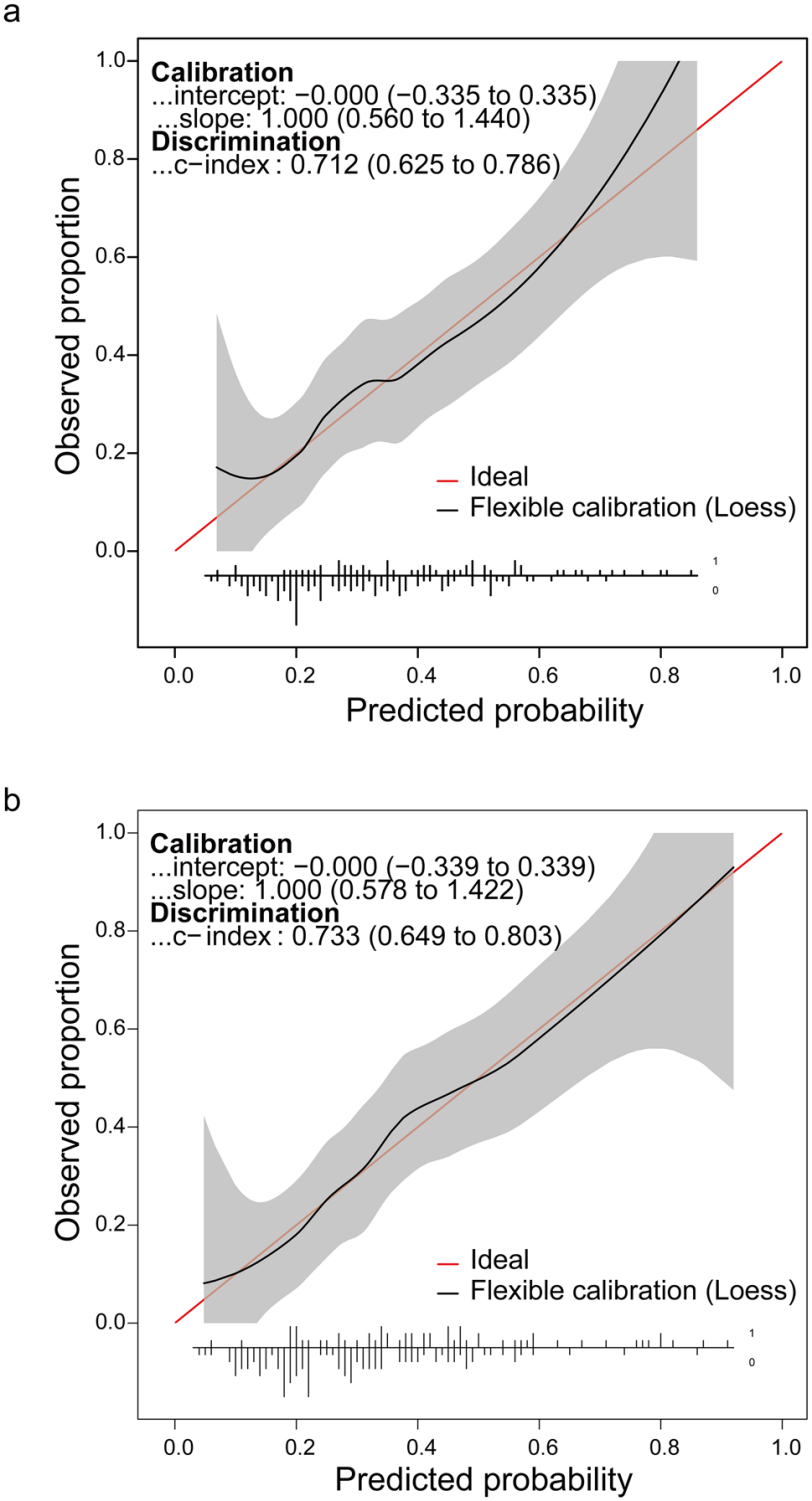
Calibration curves of nomograms estimating Parkinson’s disease risk in aged 40–80 year subjects. **a** Calibration curves of nomograms included diabetes, smoking, alcohol consumption, tea consumption, percentage of LD CD4^+^ T cells, and percentage of CD8^+^ Tn cells. **b** Calibration curves of nomograms diabetes, smoking, alcohol consumption, tea consumption, counts of LD CD4^+^ T cells, and counts of CD8^+^ Tn cells. The horizontal axis represents the nomogram-predicted probability of PD occurrence, and the vertical axis represents the actual PD diagnosis. The red line represents ideal calibration. The black line represents actual calibration, which is shaded with a 95% confidence interval. The intercept and slope of calibration curves, C-index of discrimination ability, and their 95% confidence interval are marked in the upper right corner. *C-index* concordance index, *PD* Parkinson’s disease, *CD8*^+^ *Tn cells* naïve CD8^+^ T cells, *LD CD4*^+^ *T cells* late-differentiated CD4^+^ T cells

Furthermore, we conducted calibration and discrimination to estimate the reliability and accuracy of these two nomograms for PD risk prediction in subjects aged 40–80 years. Both plotted calibration curves corresponded to the ideal plots, revealing a favorable agreement on the nomogram estimation and actual observation regarding the probability of PD risk prediction. From the distance of the actual curve to the ideal plot, we noticed that the model that included the absolute number of cells performed better than the model that included the percentage of cells. Both models showed high accuracy, with a C-index of 0.712 (95% CI 0.625–0.786) in the nomogram of cell proportion, and with a C-index of 0.733 (95% CI 0.649–0.803) in the nomogram of cell counts (Fig. 5), suggesting that these nomograms for PD risk prediction are reliable and accurate.

## Discussion

T cells play a critical role in the pathogenesis of PD [6, 25]. Since the heterogeneity and plasticity of T cells, it is promising to study the disturbed subpopulations of T lymphocytes in PD. In this study, we investigated up to 22 subsets of peripheral T lymphocytes in 175 participants using flow cytometry. We found that PD patients exhibited decreased CD8^+^ Tn cells and increased LD CD4^+^ T cells compared to HC subjects. Moreover, the proportions of CD8^+^ Tn cells and LD CD4^+^ T cells were associated with the clinical characteristics of PD. We further generated a predictive nomogram model, which reveals that both these two lymphocytes can be potential indicators of PD condition. The current work identified the key cellular components that contribute to altered immune function in PD, and laid the foundation for exploring biomarkers for disease surveillance or targeted intervention.

T cells at various differentiation stages possess distinct proliferative capacity and cytotoxicity, yet a few studies have addressed the differentiated subsets of T lymphocytes. In this study, we used CD27 and CD28 to discriminate the differentiated subsets of T lymphocytes. CD27 and CD28 are co-stimulatory molecular receptors that play an important role in antigen-specific differentiation [26, 27]. CD28^ + ^CD27^ +^ cells, which represent the early stage of T-cell differentiation, have a high proliferative capacity and a lower cytotoxic effect, while loss of the CD27 and CD28 is associated with the acquisition of the senescent phenotype [28].

Our study identified that LD CD4^+^ T cells, expressing CD3^+ ^CD4^+ ^CD27^−^ CD28^−^, increased in PD populations. Notably, increased LD CD4^+^ T cells are one of the features of senescence [29, 30], which is involved in multiple diseases, especially cytomegalovirus infection [31]. Notably, in neurodegenerative disease, late-differentiated T cells marked with CD28^−^, significantly expanded in the peripheral blood of patients with Alzheimer’s Disease (AD) [32], implying that PD and AD share a common cell pathology in immunosenescence. However, the function of LD CD4^+^ T cells remains unclear, despite mediating antigen-specific cytotoxicity against infected target cells and displaying immunosuppressive or regulatory functions in certain conditions [30], Nevertheless, our results suggest that PD is closely correlated to the aging of the adaptive immune system. Since the function of senescence T cells is reversible by the treatment of a supramolecular complex containing AMPK, TAB1, and p38 [33], the LD CD4^+^ T cells may be a potential target for PD intervention.

Previous studies have investigated functional subpopulations of T lymphocytes, such as naïve T cells [34], memory T cells [17, 34, 35], activated T cells [21], and regulatory T cells [14, 17, 19, 22, 35, 36]in the PD population. Naïve T cells, labeled with the high-molecular-weight isoform of leukocyte common antigen CD45 containing the A exon (CD45RA) [37], represent the ability of the organism to renew T lymphocytes. In our cohort, another subset that was significantly altered in PD patients was CD8^+^ Tn cell, marked with CD3^+^ CD8^+^ CD45RA^+^ CD45RO^−^. Compared to HC, PD patients showed a consistent decline of CD8^+^ Tn cells, which was also independent of the therapeutic agents. This result was in agreement with the latest studies performed in the United States and Mexico separately [38, 39], suggesting the consistent changes of CD8^+^ Tn cells in PD among different races. As the proliferation of naïve T cells and their differentiation into memory T cells are related to cellular senescence [40], the decrease of CD8^+^ Tn cells may reflect abnormal aging in PD patients.

CD45RA are replaced by the low-molecular-weight isoform CD45RO after naïve T cells activation, and then, memory T cells are formed [37]. Memory T cells respond to reinvading pathogens quickly and can be further divided into central memory T cells (Tcm) and affect memory T cells (Tem) according to their homing to different tissues [41]. Tcm cells show high proliferative capacity, while Tem cells show high killing capacity. Regulatory T cells are a subset of T cells that control autoimmune responsiveness and play an important role in maintaining the dynamic balance of the body’s immune system. However, we did not observe the significant difference in memory T cells and regulatory T cells between PD patients and the control population. The expression of CD38 and HLA-DR molecules on T lymphocytes represents the activation status of the immune cells. Chiba et al. reported that activated T cells were upregulated in PD patients [21], implying the continuous over-activation of the immune system in PD condition, which may cause immune damage and disease progression. Our study observed a similar upward trend of activated T cells in PD patients, although that change did not reach statistical significance.

The peripheral inflammatory responses correlate with the progression of PD [42–44], inferring that the pathological changes in the brain can be reflected in the blood. However, only a small number of studies were performed to investigate the changes of lymphocytes on account of the diverse clinical manifestations [20, 36, 45, 46]. In this study, we evaluated various clinical features of PD and identified independent correlations between lymphocyte subsets and PD symptoms. We then identified several non-motor factors significantly associated with CD8^+^ Tn cells, which further support the involvement of peripheral inflammation in the non-motor symptoms of PD [47]. In addition, the onset age of PD may also affect the peripheral immune system, such as LD CD4^+^ cells [48]. Our study provided candidates of cellular markers for clinical applications and potential mechanisms underlying the pathophysiology of PD.

Nomogram is a pictorial representation of statistical models constructed by clinical variables, and is proposed as a means to improve disease prognostication [49]. Recently, it has been applied for survival prediction [50] and the early diagnosis of PD [51]. Using the nomogram, we developed individualized PD risk prediction models including CD8^+^ Tn cells, LD CD4^+^ T cells, and independent risk factors such as the status of diabetes mellitus, smoking, alcohol consumption, and tea consumption. According to calibration curves, the nomogram that included absolute cells number performs better than the model included cell proportion in predicting PD risk. To the best of our knowledge, it is the first time that circulation lymphocyte subsets have been incorporated into the nomograms for PD risk prediction. Moreover, this study indicated that lymphocyte subsets in peripheral blood, especially CD8^+^ Tn cells and LD CD4^+^ T cells, are promising factors in the prediction of PD. Though we performed the discrimination and calibration, the sample size of our cohort is not large enough to validate the two nomogram models in the internal cohort and no external validation was applied either. Besides, a wide variety of exogenous factors were associated with PD risks, such as anxiety or depression, head injury, serum uric acid, and calcium channel blocker medication [52]. These known or unknown factors were not taken into consideration, which might have resulted in some bias in multivariate regression analyses. In the future, expanding the sample size and validating these nomograms in another cohort of patients at different disease stages are necessary. When possible, prospective cohort studies may be conducted to analyze the causal relationship between the alterations of two identified subsets (CD8^+^ Tn cells and LD CD4^+^ T cells) and PD.

Besides, we found that both the proportion and the absolute number of CD8^+^ Tn cells and LD CD4^+^ T cells did not differ between drug-naïve patients and drug-treated patients, suggesting that the administration of antiparkinsonian drugs does not affect the peripheral adaptive immunity, similar to the conclusion from a previous study [14]. Indeed, it is difficult to fully exclude the contribution of therapeutic agents to these cellular alterations, as medication and disease progression usually change in parallel. Rigorous prospective cohort studies are essential to clarify the association between antiparkinsonian drugs and peripheral immunity.

## Conclusion

In this study, we characterized up to 22 subpopulations of T lymphocytes in PD patients and age-matched healthy subjects. Generally, PD patients exhibit altered CD8^+^ Tn cells and LD CD4^+^ T cells in the peripheral blood, associated with their non-motor symptoms and onset age of disease. Moreover, we developed the nomogram models for PD risk prediction, which unveiled the strong relationship between T lymphocyte subsets and PD. In addition, the model that included the absolute number of cells was more reliable in predicting PD risk compared to the model that incorporated two cells percentage. These data suggest that peripheral cellular immunity is disturbed in PD patients, and the two lymphocyte subsets are important for PD prediction. Last but not least, we consider CD8^+^ Tn cells and LD CD4^+^ T cells as candidates for multicentric clinical study and underlying pathomechanism study of PD.

## Supplementary Information

Below is the link to the electronic supplementary material.

Supplementary file1 (XLSX 11 KB)

**Online Resource 2. Gating strategy used in flow cytometric analysis for evaluating the proportions of each lymphocyte subpopulations in peripheral blood. a** Dotplot of size FSC vs SSA for lymphocytes, inside P2 gate. **b** Dotplot of CD3 vs CD19, inside lymphocytes gate, where Q1-UL represents B cells (CD3^-^ CD19^+^). **c** Dotplot of CD3 vs CD(16^+ ^56), inside lymphocytes gate, where Q1-UL represents NK cells (CD3^-^ CD(16^+^ 56)^+^) and Q1-UR represents NKT cells (CD3^+ ^CD(16^+ ^56)^+^). **d** Dotplot of CD3 vs SSC, inside lymphocytes gate, where R3 represents T cells (CD3^+^). **e** Dotplot of CD57 vs CD3, inside lymphocytes gate, where U4-UR represents senescent T cells (CD3^+^ CD57^+^). **f** Dotplot of CD3 vs CD4, inside lymphocytes gate, where Q4-UR represents CD4^+^ T cells (CD3^+^CD4^+^). **g** Dotplot of CD8 vs CD3, inside lymphocytes gate, where Q2-UR represents CD8^+^ T cells (CD3^+^ CD8^+^). **h** Dotplot of CD8 vs CD28, inside lymphocytes gate, where Q1-UR represents CD8^+^ Treg cells (CD8^+^ CD28^+^). **i**, **j** The gating strategy for CD4^+^ Treg cells. **i** CD4^+^ T cells were gated by CD4 and SSC in R8. **j** Dotplot of CD127 vs CD25, inside CD4^+^ T cells gate, where Q14-UL represents CD4^+^ Treg cells (CD4^+^ CD25^+  ^CD27^-^). **k–m** The gating strategy for naïve T cells and memory T cells. **k** CD8^+^ T cells were gated by CD3 and CD8 in R1, while the gate of R5 presents CD4^+^ T cells. **l** Dotplot of CD45RA vs CD45RO, inside CD8^+^ T cells gate, where Q8-UL represents CD8^+^ Tm cells (CD3^+ ^CD8^+ ^CD45RA^- ^CD45RO^+^) and Q8-LR represents CD8^+^ Tn cells (CD3^+^ CD8^+^ CD45RA^+^ CD45RO^-^). **m** Dotplot of CD45RA vs CD45RO, inside CD4^+^ T cells gate, where Q9-UL represents CD4^+^ Tm cells (CD3^+^ CD4^+^ CD45RA^-^ CD45RO^+^) and Q9-LR represents CD4^+^ Tn cells (CD3^+ ^CD8^+^ CD45RA^+^ CD45RO^-^). **n–p** The gating strategy for Tem cells and Tcm cells. **n** CD8^+^ T cells were gated by CD3 and CD8 in R6, while the gate of R7 presents CD4^+^ T cells. **o** Dotplot of CD62L vs CD45RO, inside CD8^+^ T cells gate, where Q6-UL represents CD8^+ ^Tem cells (CD3^+^ CD8^+ ^CD45RO^+^ CD62L^-^) and Q6-UR represents CD8^+^ Tcm cells (CD3^+ ^CD8^+ ^CD45RO^+^ CD62L^+^). **p** Dotplot of CD62L vs CD45RO, inside CD4^+^ T cells gate, where Q7-UL represents CD4^+^ Tem cells (CD3^+ ^CD4^+ ^CD45RO^+ ^CD62L^-^) and Q7-UR represents CD4^+^ Tcm cells (CD3^+^ CD4^+^ CD45RO^+^ CD62L^+^). **q–s** The gating strategy for activated T cells. **q** CD8^+^ T cells were gated by CD3 and CD8 in R1, while the gate of R5 presents CD4^+^ T cells.** r** Dotplot of CD38 vs HLA-DR, inside CD8^+^ T cells gate, where Q8-UR represents activated CD8^+^ T cells (CD3^+^ CD8^+^ CD38^+^ HLA-DR^+^). **s** Dotplot of CD38 vs HLA-DR, inside CD4^+^ T cells gate, where Q9-UR represents activated CD4^+^ T cells (CD3^+^ CD4^+^ CD38^+^ HLA-DR^+^). **t–v** The gating strategy for differentiated subgroups of T cells. **t** CD8^+^ T cells were gated by CD3 and CD8 in R6, while the gate of R7 presents CD4^+^ T cells. **u** Dotplot of CD28 vs CD27, inside CD8^+^ T cells gate, where Q6-UR represents ED CD8^+^ T cells (CD3^+ ^CD8^+^ CD28^+ ^CD27^+^), Q6-LR represents MD CD8^+^ T cells (CD3^+^ CD8^+^ CD28^+^ CD27^-^) and Q6-LL represents LD CD8^+^ T cells (CD3^+^ CD8^+ ^CD28^-^ CD27^-^). **v** Dotplot of CD28 vs CD27, inside CD4^+^ T cells gate, where Q7-UR represents ED CD4^+^ T cells (CD3^+^ CD4^+^ CD28^+^ CD27^+^), Q7-LR represents MD CD4^+^ T cells (CD3^+^ CD4^+^ CD28^+ ^CD27^-^) and Q7-LL represents LD CD4^+^ T cells (CD3^+^ CD4^+^ CD28^-^ CD27^-^) (TIF 5457 KB)

**Online Resource 3. Level of leukocyte and lymphocyte in peripheral blood between groups of patients with Parkinson’s disease and healthy controls. a** Number of leukocytes. **b** Percentage of lymphocytes. **c** Number of lymphocytes. Data were presented as a box plot, with the center, box, and whiskers, corresponding to the median, interquartile range, and extremum range, respectively. *P*-values were calculated by Student’s *t*-test (variable of Lymphocyte (%)) or Mann-Whitney test (variable of Leukocyte(×10^12^/L) and Lymphocyte (×10^9^/L)). PD: *n*=115, HC: *n*=60. * indicates *P*<0.05, ** indicates *P*<0.01. *PD* Parkinson’s disease, *HC* healthy control (PDF 434 KB)

**Online Resource 4. The difference in the percentage of T lymphocyte subpopulations between drug-naïve patients and drug-treated patients with Parkinson’s disease. a** Scatter plot of percentage of CD8^+^ Tn cells and LD CD4^+^ T cells. **b** Scatter plot of the absolute number of CD8^+^ Tn cells and LD CD4^+^ T cells. Data are presented as the median and standard error of mean in the scatter plot and are compared with a Mann-Whitney test. Drug-naïve patients: *n*=25, Drug-treated patients: *n*=90. CD8^+^ Tn cells, naïve CD8^+^ T cells. *LD CD4*^*+*^* T cells* late-differentiated CD4^+^ T cells (PDF 148 KB)

Supplementary file5 (XLSX 13 KB)

Supplementary file6 (XLSX 15 KB)

## Abbreviations

PD: Parkinson’s disease
HC: Healthy controls
LEDD: Levodopa-equivalent daily dose
MDS-UPDRS: Movement Disorder Society Unified Parkinson’s Disease Rating Scale
BBS: Berg Balance Scale
Mini-BEST: Mini version of Balance Evaluation Systems Test
SPES/SCOPA: Short Parkinson’s Evaluation Scale/Scales for Outcomes in Parkinson’s disease
NMSS: Non-motor Symptom Scale
HRS: Hyposmia Rating Scale
CSI: Constipation Severity Instrument
PSQI: Pittsburgh Sleep Quality Index
RBDSQ: REM sleep Behavior Disorder Screening Questionnaire
ESS: Epworth Sleeping Scale
RLS: Restless Leg Syndrome
HAD-A/D: Hospital Anxiety and Depression Scale-Anxiety part/Depression part
HAMD-17: 17-Item Hamilton Depression Scale
MMSE: Mini-mental State Examination
SCOPA-AUT: Scales for Outcomes in Parkinson's disease for Autonomic symptoms
SCOPA-PC: Scales for Outcomes in Parkinson's disease for Psychiatric Complications
PDQ-39: 39-Item Parkinson’s Disease Questionnaire
NK: Natural killer
Tn: Naïve T cells
Tm: Memory T cells
Tcm: Central memory T cells
Tem: Effector memory T cells
ED: Early-differentiated
MD: Medium-differentiated
LD: Late-differentiated
