# Supplementary figures and images for: Changes of T lymphocyte subpopulations and their roles in predicting the risk of Parkinson’s disease
Source: J Neurol. 2022 May 24;269(10):5368–81. doi: 10.1007/s00415-022-11190-z (PMC9467943; doi:10.1007/s00415-022-11190-z)

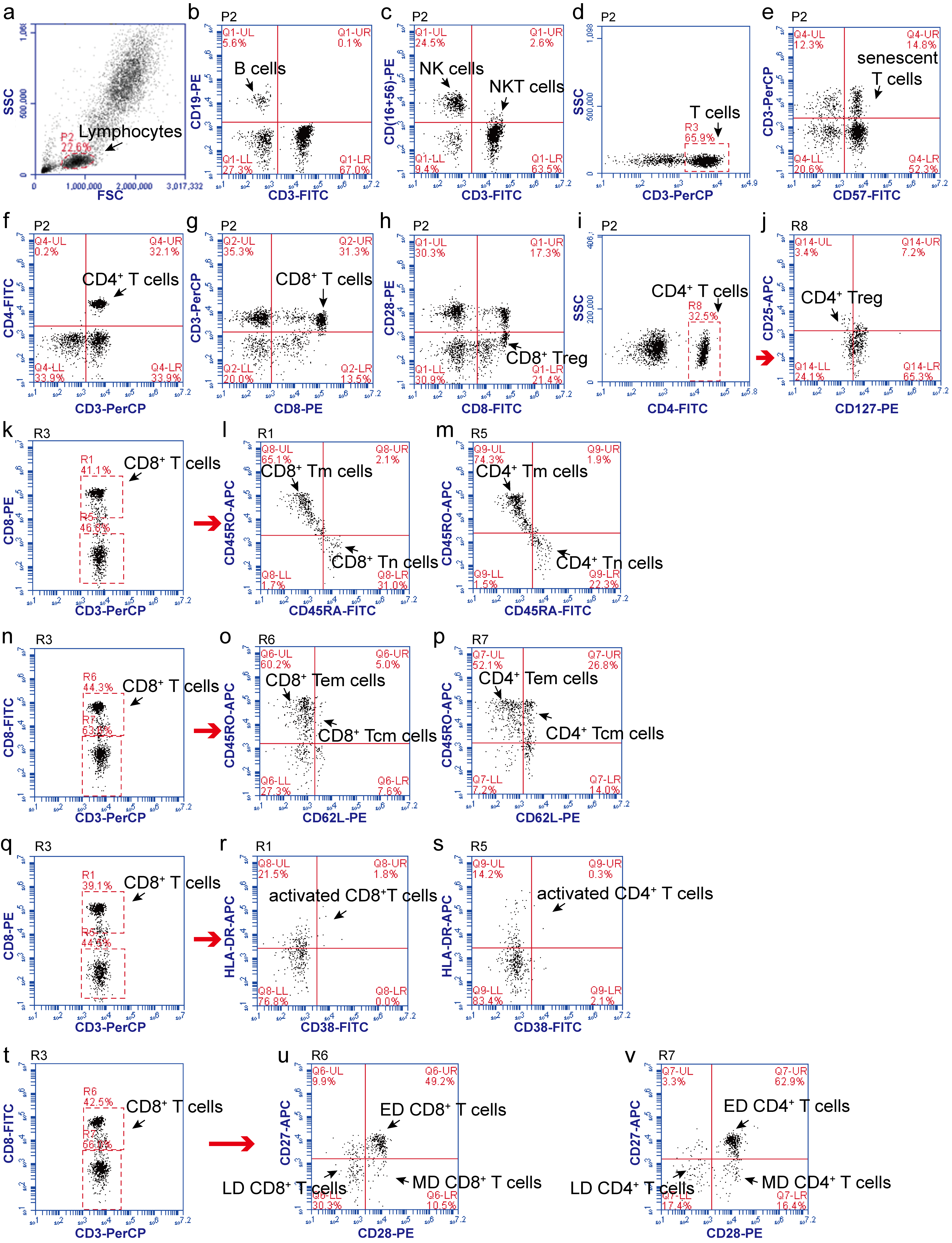

Supplement: Supplementary file 2 — Online Resource 2. Gating strategy used in flow cytometric analysis for evaluating the proportions of each lymphocyte subpopulations in peripheral blood. a Dotplot of size FSC vs SSA for lymphocytes, inside P2 gate. b Dotplot of CD3 vs CD19, inside lymphocytes gate, where Q1-UL represents B cells (CD3- CD19+). c Dotplot of CD3 vs CD(16+ 56), inside lymphocytes gate, where Q1-UL represents NK cells (CD3- CD(16+ 56)+) and Q1-UR represents NKT cells (CD3+ CD(16+ 56)+). d Dotplot of CD3 vs SSC, inside lymphocytes gate, where R3 represents T cells (CD3+). e Dotplot of CD57 vs CD3, inside lymphocytes gate, where U4-UR represents senescent T cells (CD3+ CD57+). f Dotplot of CD3 vs CD4, inside lymphocytes gate, where Q4-UR represents CD4+ T cells (CD3+CD4+). g Dotplot of CD8 vs CD3, inside lymphocytes gate, where Q2-UR represents CD8+ T cells (CD3+ CD8+). h Dotplot of CD8 vs CD28, inside lymphocytes gate, where Q1-UR represents CD8+ Treg cells (CD8+ CD28+). i, j The gating strategy for CD4+ Treg cells. i CD4+ T cells were gated by CD4 and SSC in R8. j Dotplot of CD127 vs CD25, inside CD4+ T cells gate, where Q14-UL represents CD4+ Treg cells (CD4+ CD25+ CD27-). k–m The gating strategy for naïve T cells and memory T cells. k CD8+ T cells were gated by CD3 and CD8 in R1, while the gate of R5 presents CD4+ T cells. l Dotplot of CD45RA vs CD45RO, inside CD8+ T cells gate, where Q8-UL represents CD8+ Tm cells (CD3+ CD8+ CD45RA- CD45RO+) and Q8-LR represents CD8+ Tn cells (CD3+ CD8+ CD45RA+ CD45RO-). m Dotplot of CD45RA vs CD45RO, inside CD4+ T cells gate, where Q9-UL represents CD4+ Tm cells (CD3+ CD4+ CD45RA- CD45RO+) and Q9-LR represents CD4+ Tn cells (CD3+ CD8+ CD45RA+ CD45RO-). n–p The gating strategy for Tem cells and Tcm cells. n CD8+ T cells were gated by CD3 and CD8 in R6, while the gate of R7 presents CD4+ T cells. o Dotplot of CD62L vs CD45RO, inside CD8+ T cells gate, where Q6-UL represents CD8+ Tem cells (CD3+ CD8+ CD45RO+ CD62L-) and Q6-UR represents CD8+ Tcm cells [file 415_2022_11190_MOESM2_ESM.tif]

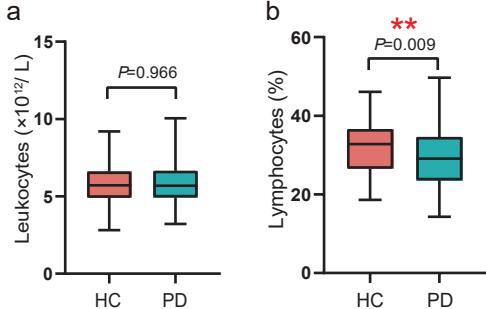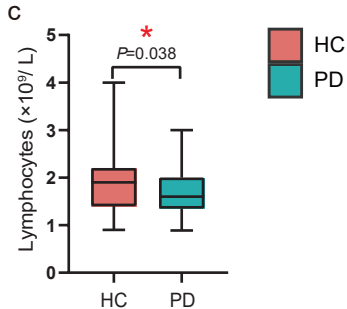

Supplement: Supplementary file 3 — Online Resource 3. Level of leukocyte and lymphocyte in peripheral blood between groups of patients with Parkinson’s disease and healthy controls. a Number of leukocytes. b Percentage of lymphocytes. c Number of lymphocytes. Data were presented as a box plot, with the center, box, and whiskers, corresponding to the median, interquartile range, and extremum range, respectively. P-values were calculated by Student’s t-test (variable of Lymphocyte (%)) or Mann-Whitney test (variable of Leukocyte(×1012/L) and Lymphocyte (×109/L)). PD: n=115, HC: n=60. * indicates P<0.05, ** indicates P<0.01. PD Parkinson’s disease, HC healthy control (PDF 434 KB) [file 415_2022_11190_MOESM3_ESM.pdf]

a

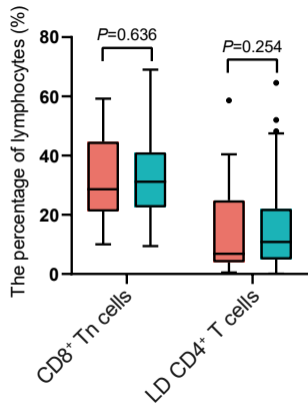

b

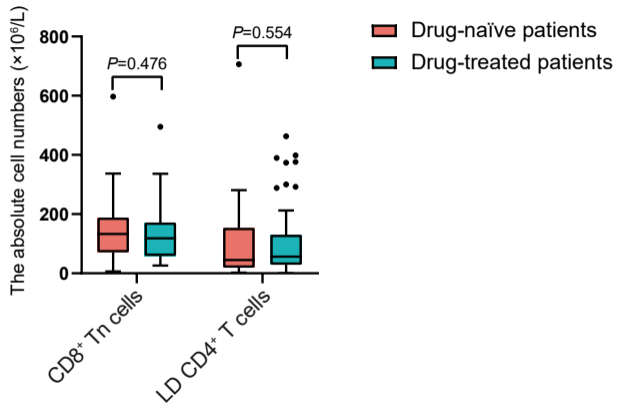

Supplement: Supplementary file 4 — Online Resource 4. The difference in the percentage of T lymphocyte subpopulations between drug-naïve patients and drug-treated patients with Parkinson’s disease. a Scatter plot of percentage of CD8+ Tn cells and LD CD4+ T cells. b Scatter plot of the absolute number of CD8+ Tn cells and LD CD4+ T cells. Data are presented as the median and standard error of mean in the scatter plot and are compared with a Mann-Whitney test. Drug-naïve patients: n=25, Drug-treated patients: n=90. CD8+ Tn cells, naïve CD8+ T cells. LD CD4+ T cells late-differentiated CD4+ T cells (PDF 148 KB) [file 415_2022_11190_MOESM4_ESM.pdf]
